# Comparison of Visual Outcomes Between Toric Intraocular Lenses and Clear Corneal Incisions to Correct Astigmatism in Image–Guided Cataract Surgery

**DOI:** 10.3389/fmed.2022.837800

**Published:** 2022-04-04

**Authors:** Ning Ding, Xudong Song, Xiaozhen Wang, Wenbin Wei

**Affiliations:** Beijing Tongren Eye Center, Beijing key Laboratory of Intraocular Tumor Diagnosis and Treatment, Beijing Ophthalmology & Visual Sciences Key Lab, Medical Artificial Intelligence Research and Verification Key Laboratory of the Ministry of Industry and Information Technology, Beijing Tongren Hospital, Capital Medical University, Beijing, China

**Keywords:** single clear corneal incision, corneal astigmatism, cataract surgery, image-guided surgery, toric IOL, Callisto eye image-guided system

## Abstract

**Purpose:**

To compare the astigmatism correction effects of toric intraocular lenses (IOL) and clear corneal incisions during image-guided cataract surgery.

**Methods:**

All patients with regular corneal astigmatism of 0.75–1.5 D underwent cataract surgery and astigmatism correction using the Callisto eye image-guided system. One group had implantation of an AcrySof toric IOL. Another group had implantation of aspheric IOL with 3.0 mm single clear corneal incision (SCCI) on the steep axis. Uncorrected and best-corrected spectacle visual acuity, refraction, and toric IOL axis were evaluated at 1, 4, and 12 weeks postoperatively.

**Results:**

Sixty-eight eyes of 68 patients were included. The mean residual refractive cylinder was 0.34 ± 0.40 D in the toric group and 0.64 ± 0.57 D in the SCCI group. There were no significant differences in residual refractive cylinder, spherical equivalent, uncorrected distance visual acuity (UDVA), and best-corrected spectacle visual acuity (BCSVA) between groups. The percentage of the residual cylinder within ± 0.50 D was 75 and 56% for toric and SCCI cases, respectively (*p* > 0.1). The mean surgical induced astigmatism vector was 0.61 ± 0.29 D in the SCCI group and 1.04 ± 0.38 D in the toric group. The mean magnitude of error was negative (−0.54 ± 0.48 D) and the correction index was <1.0 (*p* < 0.05) in SCCI group. At 3 months, all toric IOL alignment errors were within 5 degrees from the intended axis.

**Conclusions:**

Both toric IOL and SCCI can correct low and medium astigmatism effectively with the help of a precise image-guided system.

## Introduction

Corneal astigmatism is one of the important factors affecting visual quality after cataract surgery. It is estimated that 67.7% of eyes had corneal astigmatism between 0.25 and 1.25 diopters (D), and 27.5% of eyes had astigmatism at 1.25 D or higher in the cataract population (1). Another study showed that corneal astigmatism in the range of 0.50–0.99 D was the most common (30.08%), followed by 1.00–1.49 D (22.15%) (2). A simple, accurate, effective, and safe method to correct astigmatism is the pursuit of surgeons.

Preoperative marking is an important step in astigmatism correction, whether using toric intraocular lenses (IOLs) or corneal incisions. Previous studies have usually used conventional manual marking with an ink pen. However, the application of an intraoperative image-guided system can improve the accuracy of IOL alignment and incision location. It has been shown that digital marking is more reliable than manual marking using a slitlamp (3). Therefore, we compared the astigmatism-reducing effect during Callisto eye image-guided cataract surgery using toric IOLs or non-toric IOL combined with 3.0 mm single clear corneal incision (SCCI) on the steep meridian in the correction of low-to-moderate regular corneal astigmatism.

## Patients and Methods

The study was approved by the Ethics Committee of Beijing Tongren Hospital, Capital Medical University, and conforms to the tenets of the Declaration of Helsinki (as revised in 2013) and good clinical practice.

A total of 68 eyes with cataracts and preoperative anterior corneal astigmatism with optical biometry (IOL Master 700, Carl Zeiss Meditec AG, Jena, Germany) of 0.75–1.5 D were enrolled in the study. The inclusion criteria were as follows: regular and symmetric astigmatism shape on the corneal topographic map, pupil dilation >6.00 mm, and no obvious ocular and systemic diseases. The exclusion criteria were as follows: undergoing pterygium surgery within 1 month, a history of intraocular surgery, irregular corneal astigmatism (corneal scar, corneal degeneration, keratoconus), and other ocular diseases (lens subluxation, uveitis, glaucoma, traumatic cataract, retinopathy, macular disease, or optic neuropathy).

**Figure 1:**
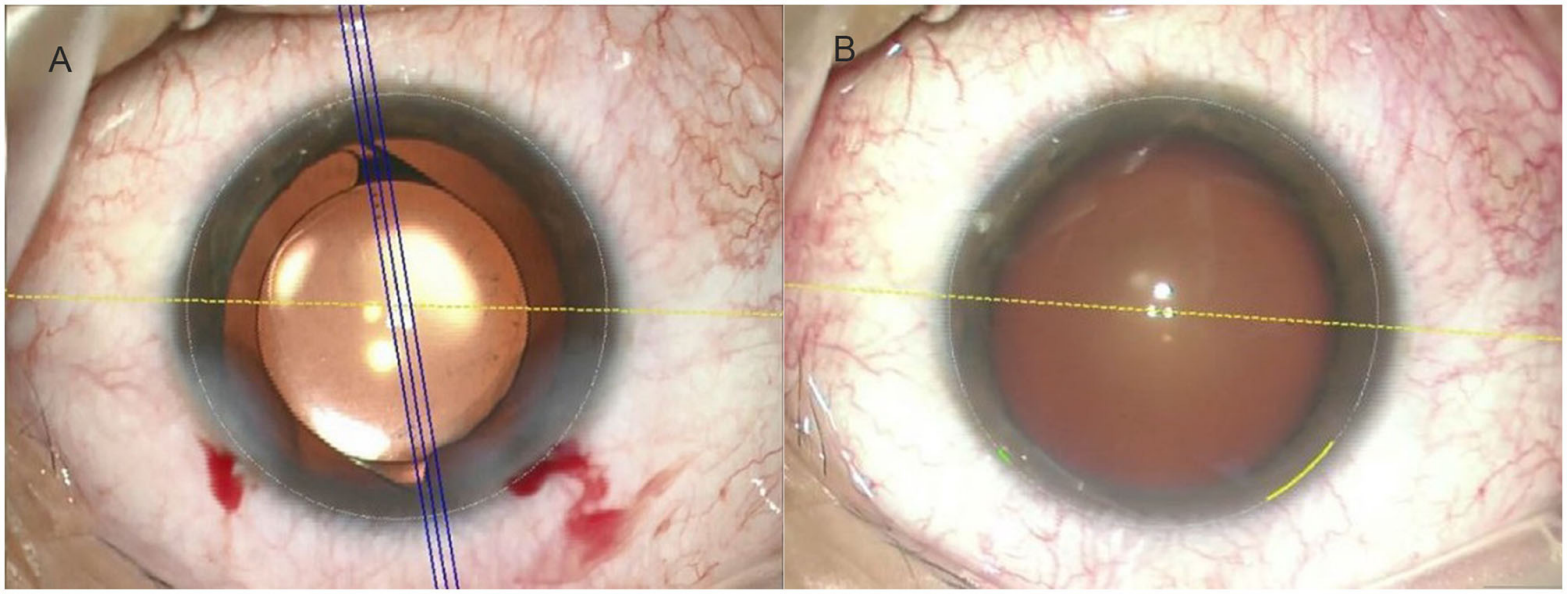
The Callisto eye image-guided system was used to determine digital markers with the Lumera microscope. **(A)** The toric intraocular lens (IOL) target axis (3 parallel blue lines indicate the intended axis, and the yellow dots indicate a 0–180-degree axis). **(B)** The yellow arc indicates a corneal incision of the steep meridian with a length of 3.0 mm.

All included patients underwent phacoemulsification and IOL implantation for astigmatism correction, including 36 eyes with toric IOL implantation and 32 eyes with aspheric monofocal IOL implantation with corneal astigmatic incisions. In the toric group, AcrySof Toric IOL (Alcon Laboratories, Inc., Fort Worth, TX, USA) power and orientation were calculated using the Barrett toric calculator (http://calc.apacrs.org/toric_calculator20/Toric%20Calculator.aspx). A 2.4 mm clear corneal incision was made on a 160° axis and surgical induced astigmatism vector (SIA) was calculated as 0.3. In the SCCI group, a 3.0 mm clear corneal incision was made at 1 mm inside limbus on the steep meridian. The IOL implanted was a MI60 (Bausch and Lomb, USA). Both groups' biometry data were obtained by IOL Master 700 and exported into the Callisto eye system (version 3.5.1.116555, Carl Zeiss Meditec AG). Results of the above calculations were preset in the Callisto eye system and the intraoperative overlay was displayed under OPMI Lumera 700 microscope (Carl Zeiss Meditec AG, Germany) to serve as a guide for the surgeon of toric IOL intended axis for the toric group and position and size of incision for SCCI group (Figure 1). All surgeries were performed by the same experienced surgeon. No complications occurred.

**Figure 2:**
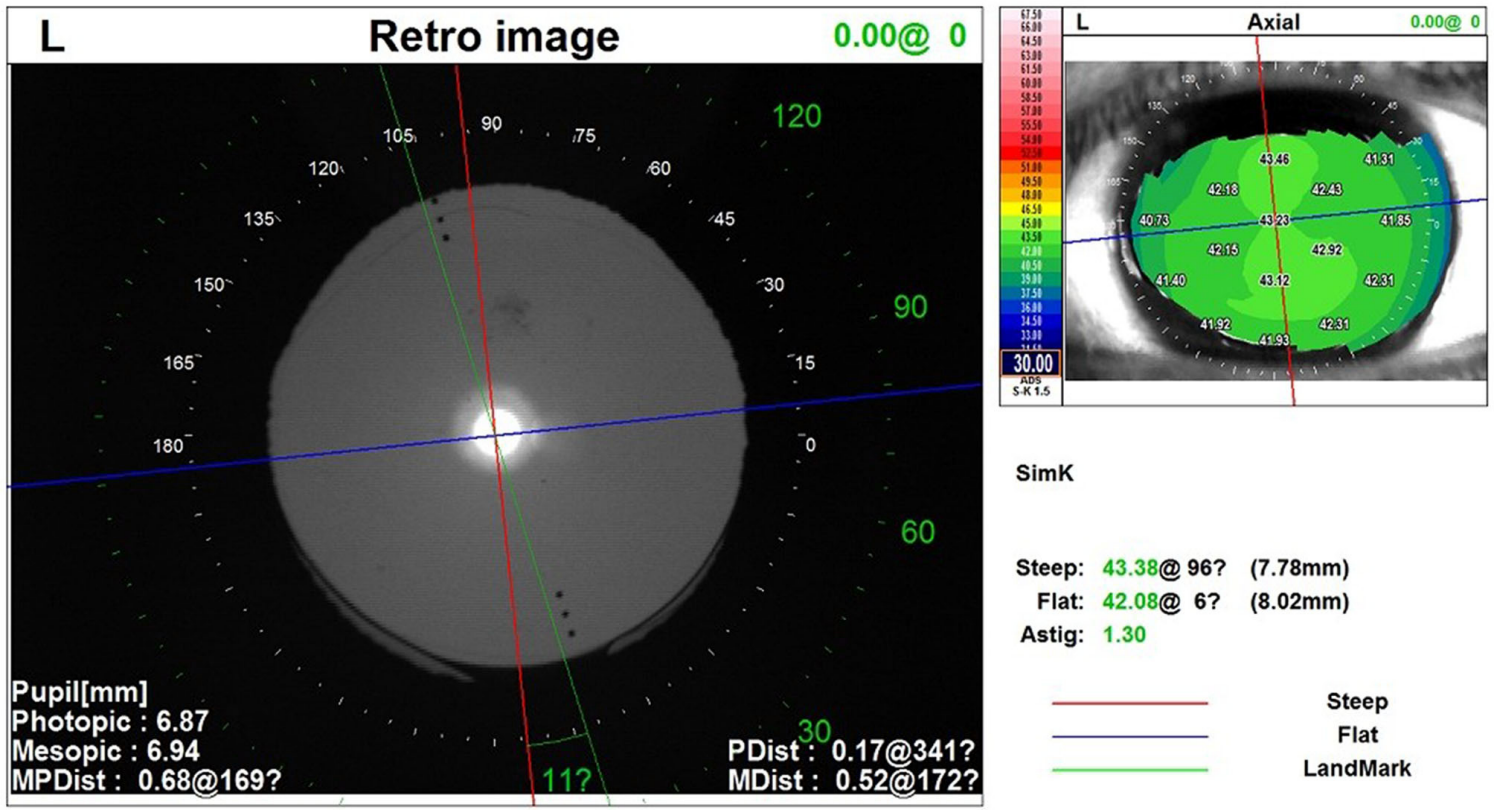
The OPD scan III was used to evaluate the toric IOL orientation using the retro image. The red line indicates the steep axis of the cornea and the blue line indicates the flat axis. The green line indicates the toric IOL orientation. The included angle degrees are displayed between the red and green lines. IOL, intraocular lens.

Participants were evaluated preoperatively and followed up 1 day, 1 week, 1 month, and 3 months postoperatively. Preoperative assessment included uncorrected distance visual acuities (UDVA), slitlamp examination, and intraocular pressure. A comprehensive evaluation of IOL Master 700, pentacam HR (Oculus Optikgerate GmbH, Wetzlar, Germany) and OPD scan III (Nidek Inc., Tokyo, Japan) was made to determine the regularity of the cornea and the suitability of toric IOL. Patients with regular central corneal topography and similar results of these three examinations were considered suitable for toric IOL implantation. Comparing the results of three examinations, if the difference of steep axis was greater than 10° or if the difference between simulated keratometry (SimK) and total corneal refractive power (TCRP) was >0.75D, then it was considered that the cornea is not regular and excluded from the study. This same process was repeated for the SCCI group. The UDVA, manifest refraction, best-corrected spectacle visual acuity (BCSVA), and toric IOL orientation were recorded at each postoperative visit. Among these, the toric IOL orientation was measured at the retro image by OPD scan III at every follow-up (Figure 2).

The residual refractive astigmatism, spherical equivalent (SE) refraction, UDVA, and BCSVA were compared in both groups at 3 months after surgery. The toric IOL orientation (intended vs. actual) at 1 and 3 months postoperatively were also evaluated.

The vector analysis of astigmatic correction was performed using the Alpins method (4, 5). The refractive astigmatism values were converted to the corneal plane for calculation. All statistical analyses were performed by Excel file (Microsoft Corp., Redmond, WA, USA) and SPSS software (version 22.0.0.0, IBM Corp., Armonk, NY, USA). *T*-test or chi-square (χ^2^) test was used for the difference between the groups when appropriate. A *p*-value of < 0.05 was considered statistically significant.

## Results

The statistical characteristics of patients at the preoperative stage and 3 months postoperatively are shown in Table 1. Preoperative astigmatism in the eyes was measured with the optical biometer. There were 72.22% with the rule (WTR) (26 eyes), 25% against the rule (ATR) (9 eyes), and 2.78% Oblique (OB) (1 eye) eyes in the toric group and 43.75% WTR (14 eyes), 50% ATR (16 eyes), and 6.25% OB (2 eyes) eyes in the SCCI group. At 3 months after surgery, the mean residual refractive cylinder was 0.34 ± 0.40 D (0–1.00 D) in the toric group and 0.64 ± 0.57 D (0–1.25 D) in the SCCI group. The mean residual astigmatism in the toric group was ~0.3 D lower than that of SCCI group, but with no difference between the 2 groups (*p* = 0.24). The mean SE refraction was 0.17 ± 0.28 D (−0.21 to 0.59 D) in the toric group and 0.13 ± 0.45 D (−0.43 to 0.90 D) in the SCCI group (*p* = 0.83). At 3 months, the average UDVA was 0.17 ± 0.22 logarithm of the minimum angle of resolution (logMAR) (0 to 0.52 logMAR) in the toric group and 0.12 ± 0.11 logMAR (−0.08 to 0.30 logMAR) in SCCI group (*p* = 0.57, *t*-test of independent samples). The mean BCSVA was 0.04 ± 0.09 logMAR (−0.08 to 0.22 logMAR) in the toric group and 0.03 ± 0.07 logMAR (−0.08 to 0.10 logMAR) in SCCI group (*p* = 0.92, *t*-test of independent samples).

**Table 1:** Comparison of outcomes before and 3 months after surgery (mean ± SD).

	**Toric IOL group**	**SCCI group**	***P*** **value**
Age (y) (range)	65.00 ± 8.03 (46 to 71)	59.22 ± 13.80 (32 to 82)	0.32
Gender (M/F)	13/23	10/22	–
Eyes (R/L)	16/20	17/15	–
Axial length (mm) (range)	23.64 ± 0.82 (22.34 to 24.47)	24.27 ± 1.05 (22.38 to 25.59)	0.19
Preop corneal cylinder (D)	1.28 ± 0.18	1.15 ± 0.27	0.26
WTR	26	14	–
ATR	9	16	–
OB	1	2	–
Keratometry 1 (range)	43.21 ± 1.16 (41.97 to 45.56)	44.09 ± 1.59 (42.1 to 47.47)	0.22
Keratometry 2 (range)	44.49 ± 1.16 (43.27 to 46.74)	45.24 ± 1.64 (42.91 to 48.76)	0.30
Residual refractive cylinder (D) (range)	0.34 ± 0.40 (0.00 to 1.00)	0.64 ± 0.57 (0.00 to 1.25)	0.24
SE refraction (D) (range)	0.17 ± 0.28 (−0.21 to 0.59)	0.13 ± 0.45 (−0.43 to 0.90)	0.83
Preop UDVA (logMAR) (range)	0.55 ± 0.38 (0.15 to 1.30)	0.87 ± 0.70 (0.22 to 2.00)	0.26
Postop UDVA (logMAR) (range)	0.17 ± 0.22 (0.00 to 0.52)	0.12 ± 0.11 (−0.08 to 0.30)	0.57
Postop BCSVA (logMAR) (range)	0.04 ± 0.09 (−0.08 to 0.22)	0.03 ± 0.07 (−0.08 to 0.10)	0.92

*The clear corneal incision was made on the steep axis. Range is minimum to maximum points*.

*SD, standard deviation; D, diopters; Toric IOL, toric intraocular lens; SCCI, single clear corneal incision. ATR, against the rule; WTR, with the rule; OB, Oblique; SE, spherical equivalent; UDVA, uncorrected distance visual acuity; BCSVA, best-corrected spectacle visual acuity*.

Table 2 lists the toric IOL models implanted in surgery.

**Table 2:** Toric IOLs power at corneal plane.

**IOL model**	**Cylinder power (D)**	**Number (%)**
SN6AT2	0.69	4 (11.11)
SN6AT3	1.03	21 (58.33)
SN6AT4	1.55	11 (30.56)

*IOL, intraocular lens*.

**Figure 3:**
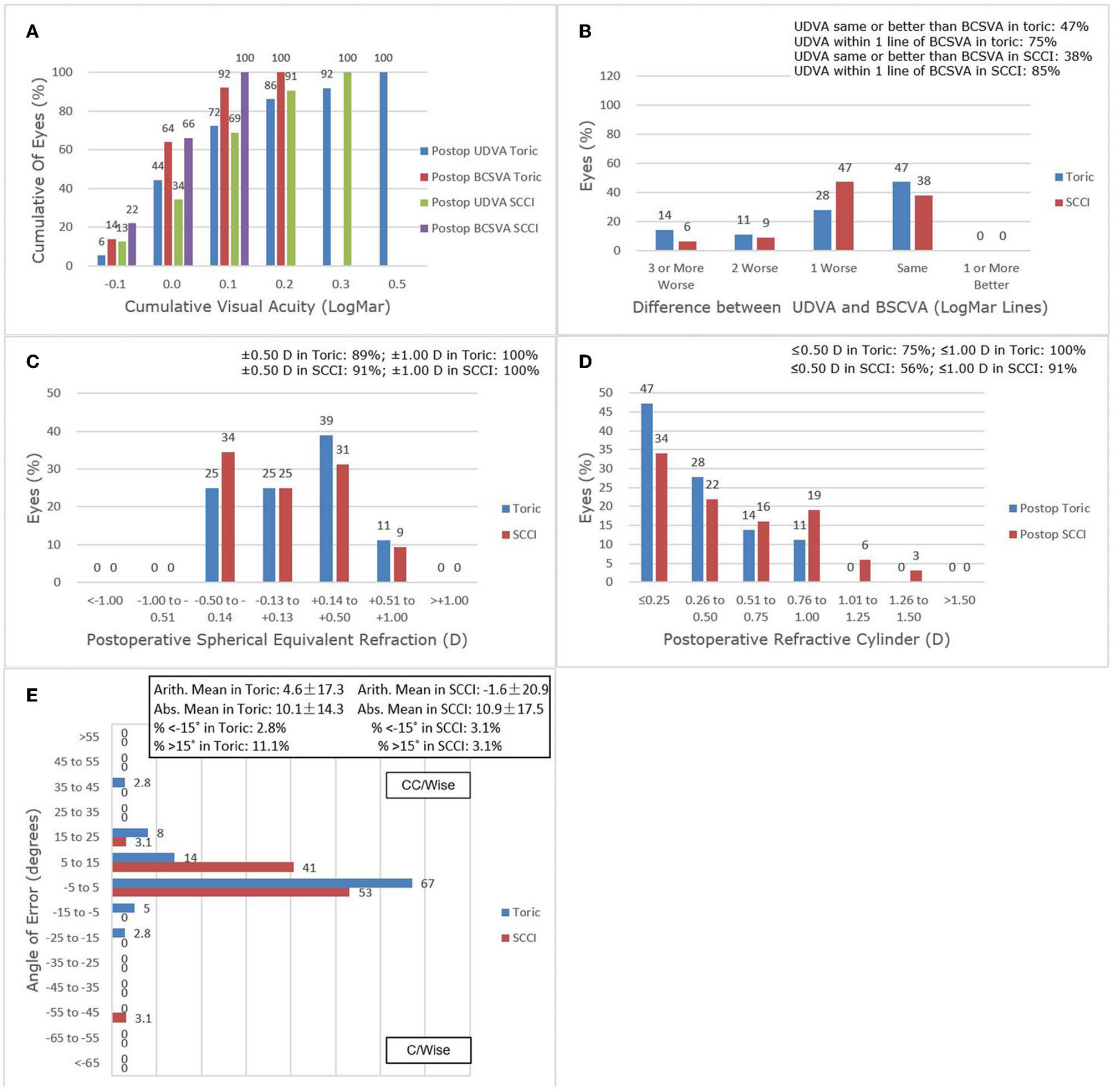
Refractive outcomes at 3 months postoperatively. **(A)** Uncorrected distance visual acuity. **(B)** Uncorrected distance visual acuity vs. best-corrected spectacle visual acuity. **(C)** Spherical equivalent refraction accuracy. **(D)** Postoperative refractive cylinder. **(E)** Refractive Astigmatism Angle of Error.

The Standard Graphs for Cataract Surgery are used to show refractive outcomes at 3 months after image-guided cataract surgery in Figure 3. The percentages of postoperative UDVA and postoperative BCSVA were significantly improved in both groups. For UDVA, 92% of toric cases and 100% of SCCI cases were < 0.3 logMAR (*p* = 0.24). For BSCVA, 92% of toric cases and 100% of SCCI cases were < 0.1 logMAR (*p* = 0.24) (Figure 3A). In postoperative UDVA, about 47% of eyes in the toric group and 38% in the SCCI group were in the same lines as BCSVA, while 75% in the toric group and 85% in the SCCI group were within 1 line of BCSVA (Figure 3B). About 89% of the toric cases and 91% of the SCCI cases were within ± 0.50 D (*p* = 1.00) in postoperative SE refraction, and all eyes in the two groups were within ± 1.00 D (Figure 3C). About 75% of toric cases and 56% of the SCCI cases were within ± 0.50 D in the residual refractive cylinder (χ^2^ = 2.661, *p* = 0.103). All toric cases were within ± 1.00 D, with the difference not being statistically significant (*p* = 0.10) (Figure 3D). Angle-of-error analysis for refraction showed that the AE (angle of error) of most eyes in both groups was between −5 and 15 degrees. The arithmetic mean was 4.6 degrees counterclockwise (CCW) in the toric group and −1.6 degrees slightly clockwise (CW) in the SCCI group, while the absolute means were 10.1 degrees in the toric group and 10.9 degrees in the SCCI group (Figure 3E; Table 3).

**Table 3:** Vector analysis for treatment and error at 3 months after surgery (mean ± SD).

	**Toric IOL group**	**SCCI group**	***P*** **value**
TIA, D (range)	1.02 ± 0.23 (0.78 to 1.31)	1.15 ± 0.27 (0.81 to 1.50)	0.320
SIA, D (range)	1.04 ± 0.38 (0.40 to 1.59)	0.61 ± 0.29 (0.31 to 0.96)	0.02
DV, D (range)	0.34 ± 0.39 (0 to 0.98)	0.62 ± 0.56 (0 to 1.46)	0.25
AE, degrees			
arithmetic mean	4.63 ± 17.25	−1.56 ± 20.93	0.52
(range)	(−22 to 39)	(−56 to 12)	
absolute mean	10.13 ± 14.32	10.89 ± 17.53	0.92
(range)	(0 to 39)	(0 to 56)	
ME, D (range)	0.02 ± 0.22 (−0.40 to 0.43)	−0.54 ± 0.48 (−1.19 to 0)	0.01
CI (range)	1.00 ± 0.24 (0.50 to 1.37)	0.58 ± 0.35 (0.21 to 1.00)	0.01
IOS (range)	0.39 ± 0.47 (0 to 1.26)	0.48 ± 0.41 (0 to 1.12)	0.67

*Range is minimum to maximum points*.

*SD, standard deviation; TIA, Target induced astigmatism; SIA, Surgically induced astigmatism; DV, Difference vector; AE, angle of error; ME, magnitude of error; CI, correction index; IOS, index of success*.

**Figure 4:**
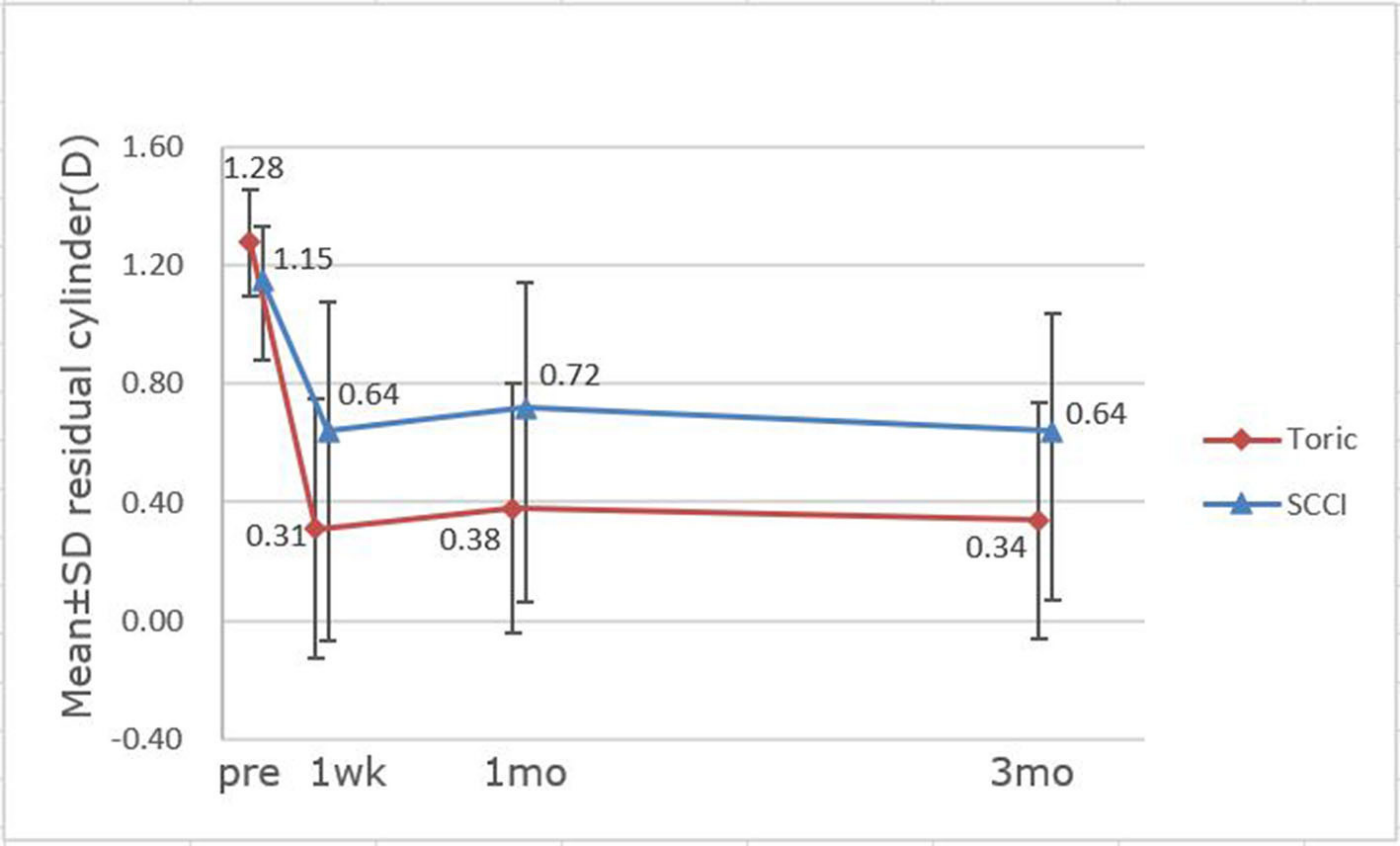
Astigmatism changes over time between toric IOL and single clear corneal incision (SCCI) groups.

Figure 4 shows preoperative corneal astigmatism and residual postoperative refractive astigmatism for each group over 3 months. The proportion of astigmatism reduction would be an average of 73.44 and 44.35% for the toric and SCCI, respectively, at 3 months after surgery.

The vector analysis results using the Alpins method are shown in Table 3. The mean SIA in SCCI group (0.61 ± 0.29 D) was less than in the toric group (1.04 ± 0.38 D) (*p* < 0.05), and it was lower than its target induced astigmatism vector (TIA) (1.15 ± 0.27 D), indicating under correction. The mean magnitude of error (ME) in the toric group was closer to 0, while the negative value (−0.54 D) in the SCCI group indicates under correction (*p* < 0.05). The correction index (CI) is preferably 1.0, but it was <1.0, which also confirmed that there was an under correction in SCCI group (*p* < 0.05). The results in the difference vector (DV) were not large in both toric (0.34 ± 0.39 D) and SCCI (0.62 ± 0.56 D) cases. The best result for index of success (IOS) is 0, and it was less in the toric group (IOS = 0.39) than in the SCCI group (IOS = 0.48). There were no statistically significant differences in TIA, DV, angle of error (AE), and IOS between the two groups.

The toric IOL orientation (intended vs. actual) was evaluated by OPD scan III and changes are shown in Table 4, including the changes at the time of surgery and 3 months postoperatively,as well as the changes from 1 to 3 months after surgery. The absolute difference of all toric IOLs from the intended axis was within 5 degrees until 3 months after surgery. No eye underwent a secondary alignment to reorient the IOL.

**Table 4:** Toric intraocular lens alignment error changes over time.

**Change**	**1 month to 3 months**	**Surgery to 3 months**
0 to 2 degrees (eyes)	32	19
3 to 5 degrees (eyes)	4	17
6 to 10 degrees (eyes)	0	0
>10 degrees (eyes)	0	0
Mean ± SD (degrees)	−0.63 ± 1.85	−0.50 ± 3.12
Median (degrees)	0	−1
Range (degrees)	−5,1	−5,4

*Range is minimum to maximum points*.

*SD, standard deviation*.

## Discussion

Modern cataract surgery brings expectations of clearer vision, greater visual quality, and less dependence on spectacles. Meanwhile, more attention has been paid to the necessity of astigmatism correction. Mild astigmatism can cause significantly decreased vision, even as low as 1.00 D. If not corrected, it has a significant effect on patients' independence, quality of life, and well being (6). Postoperative residual astigmatism of < 0.5 D is recommended to achieve better visual function and patient satisfaction after cataract surgery. However, how to suitably correct astigmatism during surgery is a big challenge for ophthalmologists.

There are various ways to correct astigmatisms in cataract surgery, such as toric IOL implantations (7, 8), astigmatic keratotomy (9), limbal relaxing incisions (LRI) (10), SCCI, or opposite clear corneal incision (OCCI) on the steep meridian (11–13), excimer laser *in situ* keratomileusis (14), and photorefractive keratectomy (15). Surgeons need to choose appropriate methods according to the amount of corneal astigmatism and the equipment of the operating room.

Toric IOLs have been widely used in cataract patients with regular astigmatism over the past few years, with good effectiveness and predictability especially in the effective correction method of medium and high astigmatism (16, 17). However, it is possible that due to inaccurate marking and the rotation of toric IOL (18), a second intraocular procedure may have to be performed to reposition the IOL, increasing the risk for infection. As a step of cataract surgery, SCCI is a simple technique that requires no additional skills or equipment. It is an easy, safe, and inexpensive method for astigmatic correction that is effective for low to moderate astigmatism. It has been reported that the size, shape, and location of a clear corneal incision (CCI) can affect corneal astigmatism (14). Corneal factors can also affect astigmatism correction, such as the size and meridian of preoperative corneal astigmatism (19), thickness and elasticity of cornea, and the extent of incision scarring after surgery (11). The main disadvantage of CCI is that it is difficult to predict accurately and the long-term correction effect may decrease. However, previous studies showed that surgically induced astigmatism was stable for a long time after operation in 3.0 mm SCCI and OCCI cases. Nemeth et al. (12) observed that the amount of astigmatism reduction is not related to the position of incisions and its effect remains unchanged during the postoperative period in the SCCI and OCCI cases. Other studies have revealed that the average astigmatism corrected by CCI may remain stable for 12 weeks (20) or even 1 year (21) after surgery.

It is widely known that accurate alignment of toric IOL is crucial for astigmatism correction, and the location of the corneal incision is the same. Precise preoperative marking is the basis of exact alignment. With the help of new technologies, the preoperative marking procedure is simplified and the patient's discomfort is greatly alleviated. Meanwhile, the astigmatism-reducing effect is improved. The image-guided system is objective and easy to use. Without requiring subjective estimation and contact with the patient's eyes during the whole surgery, it can project real-time digital image guidance on the eye to identify the target meridian on the operating microscope, reducing the patient's psychological and eye discomfort. A prospective study in India showed that using the slit-lamp marking method about 28% of toric cases had an alignment error of more than 5 degrees (17). Another study showed that marking under a slit lamp using a marker pen or toric marker caused an average axis misalignment of 3.4 to 6.9 degrees. As a result, the astigmatism correction effect is reduced by 10 to 20% on average (22). Several image-guided modalities have been used in clinical practice for precise and contactless alignment in order to decrease the subjectivity of manual marking (3, 23–25) and the technical dependence on the operator. Research has shown that image-guided marking is superior to manual marking, with more precise alignment, less axial misalignment, and better refractive outcomes (23, 24, 26). Other studies have found that although visual acuity is similar between the image-guided group and manual group, the former has better visual quality and the difference is clinically significant (27). Moreover, both the mean toric IOL alignment time and total operation time are significantly shorter in the digital group (23).

We compared toric IOLs with 3.0 mm SCCI. The results showed that the mean residual astigmatism of the toric group was ~0.3 D less than that of SCCI, but with no difference between the 2 groups (*p* >0.05). With the corneal wound healing process, we found that residual astigmatism was postoperatively stable in both groups over 3 months. The residual refractive cylinder was 0.64 ± 0.57 D on average in the SCCI group at 3-month follow-up, which was slightly lower than the finding of previous research. Ren et al. reported the mean corneal astigmatism was reduced to 0.82 ± 0.68 D in 3.0 mm SCCI group at 3 months after surgery (28). Though it has been shown that OCCI is better than SCCI of the same size (28) in reducing astigmatism, OCCI adds one corneal incision, prolongs the operation time, and has greater potential damage to the cornea. In the current study, there were no significant differences in the residual refractive cylinder, SE, UDVA, and BCSVA between the groups. The proportion of residual astigmatism within ± 0.5 D was higher in the toric IOL group compared with SCCIs (*p* >0.1). As is well known, the effect of posterior corneal astigmatism on postoperative manifest refractive astigmatism would differ according to the meridian of the anterior steep axis. This will reduce with the rule astigmatism and increase against the rule astigmatism. The proportion of WTR in the toric group (72.22%) was higher than that of SCCI group (43.75%). Hence, it is possible to underestimate the astigmatism reduction in the SCCI group. Furthermore, our results showed that all of the toric IOL alignment errors were within 5 degrees from the intended axis at 3 months, and the mean error in alignment was −0.50 ± 3.12 degrees. This alignment error is lower than what is reported in other studies. Farooqui et al. (16) showed that 6% of toric cases had a misalignment of more than 10 degrees by slit-lamp method. Webers et al. (24) found that the mean misalignment of toric IOL was 1.7 ± 1.5 degrees in the image-guided group at 3 months. Emesz et al. (29) stated that less effective correction in the low toric IOL group may be caused by slight misalignment and measurement errors. However, our findings suggest that by using a new digital navigation technique, the alignment of the IOL during surgery is more accurate. Accurate alignment, skilled surgical technique, and good IOL rotation stability will bring the better effect of astigmatism correction.

Meanwhile, we performed vector analysis by Alpins method. It can be seen that the correction effect of the toric group is better, while that of SCCI group is slightly under corrected (Table 3). In the current study, as the SCCI group cannot accurately predict TIA like the toric group. For the convenience of calculation, TIA of the SCCI group was set as full correction for calculation, possibly causing errors and affecting the statistical results. In addition, there are other factors at work, such as posterior corneal astigmatism. However, the trend of under correction for the SCCI group is evident. IOS suggested that the postoperative astigmatic status was better in the toric group (0.39 = 61%) than in the SCCI group (0.48 = 52%), but the difference was not statistically significant.

Moreover, the image-guided system had some limitations. Although the computer-assisted markerless system provided better outcomes than using manual marking, it should be noted that the intraoperative factors (e.g., conjunctival edema or hemorrhages) might affect the real-time identification of limbal and scleral vessels, resulting in deviation either at the beginning of the procedure or during the operation. Sometimes, anterior segment photos of sufficient quality were not available by IOL Master 700 due to dry eyes or poor coordination. These patients still need to be manually marked and excluded from the study.

In summary, combined use of 3.0-mm SCCIs on the steep meridian with the Callisto eye image-guided system can effectively correct mild to moderate corneal astigmatism in cataract surgery. In eyes with up to 1.50 D of regular corneal astigmatism, according to respective surgical conditions, both 3.0 mm SCCIs or toric IOL implantations can be selected combined with accurate alignment, which can achieve a good effect of astigmatic correction at the time of cataract surgery.

## Data Availability Statement

The original contributions presented in the study are included in the article/supplementary materials, further inquiries can be directed to the corresponding author/s.

## Ethics Statement

This retrospective study was approved by the Ethics Committee of Beijing Tongren Hospital, Capital Medical University (TRECKY2020-124). Written informed consent for participation was not required for this study in accordance with the national legislation and the institutional requirements.

## Author Contributions

ND designed the study, examined patients, analyzed and interpreted results, and wrote the manuscript. XS performed the cataract surgery with intraocular lens (IOL) implantation and astigmatism correction and reviewed the manuscript. XW analyzed results. WW reviewed the manuscript. All authors contributed to the article and approved the submitted version.

## Funding

This study was supported by the Capital Health Research and Development of Special (2020-1-2052), Science and Technology Project of Beijing Municipal Science, and Technology Commission (Z201100005520045 and Z181100001818003). The funding bodies had no role in the manuscript.

## Conflict of Interest

The authors declare that the research was conducted in the absence of any commercial or financial relationships that could be construed as a potential conflict of interest.

## Publisher's Note

All claims expressed in this article are solely those of the authors and do not necessarily represent those of their affiliated organizations, or those of the publisher, the editors and the reviewers. Any product that may be evaluated in this article, or claim that may be made by its manufacturer, is not guaranteed or endorsed by the publisher.
